# Mapping Portuguese Natura 2000 sites in risk of biodiversity change caused by atmospheric nitrogen pollution

**DOI:** 10.1371/journal.pone.0198955

**Published:** 2018-06-21

**Authors:** Pedro Pinho, Teresa Dias, Cláudia M. d. S. Cordovil, Ulrike Dragosits, Nancy B. Dise, Mark A. Sutton, Cristina Branquinho

**Affiliations:** 1 cE3c, Centre for Ecology, Evolution and Environmental Changes, Faculdade de Ciências, Universidade de Lisboa, Lisboa, Portugal; 2 CERENA, Centro de Recursos Naturais e Ambiente, Instituto Superior Técnico, Universidade de Lisboa, Portugal; 3 LEAF, Instituto Superior de Agronomia, Universidade de Lisboa, Tapada da Ajuda, Lisboa, Portugal; 4 NERC Centre for Ecology & Hydrology (CEH), Edinburgh Research Station, Bush Estate, Penicuik, Midlothian, United Kingdom; Trent University, CANADA

## Abstract

In this paper, we assess and map the risk that atmospheric nitrogen (atN) pollution poses to biodiversity in Natura 2000 sites in mainland Portugal. We first review the ecological impacts of atN pollution on terrestrial ecosystems, focusing on the biodiversity of Natura 2000 sites. These nature protection sites, especially those located within the Mediterranean Basin, are under-characterized regarding the risk posed by atN pollution. We focus on ammonia (NH_3_) because this N form is mostly associated with agriculture, which co-occurs at or in the immediate vicinity of most areas of conservation interest in Portugal. We produce a risk map integrating NH_3_ emissions and the susceptibility of Natura 2000 sites to atN pollution, ranking habitat sensitivity to atN pollution using expert knowledge from a panel of Portuguese ecological and habitat experts. Peats, mires, bogs, and similar acidic and oligotrophic habitats within Natura 2000 sites (most located in the northern mountains) were assessed to have the highest relative risk of biodiversity change due to atN pollution, whereas Natura 2000 sites in the Atlantic and Mediterranean climate zone (coastal, tidal, and scrubland habitats) were deemed the least sensitive. Overall, results allowed us to rank all Natura 2000 sites in mainland Portugal in order of evaluated risk posed by atN pollution. The approach is of great relevance for stakeholders in different countries to help prioritize site protection and to define research priorities. This is especially relevant in countries with a lack of expertise to assess the impacts of nitrogen on biodiversity and can represent an important step up from current knowledge in such countries.

## Introduction

### Atmospheric N pollution

Nitrogen (N) pollution is a major environmental challenge [1, 2]. Without the use of N-containing fertilizers, the human population would have been approximately half of its current size [1], but 50–75% of the N applied in agriculture is not taken up by crops. This excess is lost to the environment, affecting human health, air, water, soil, climate, and ecosystems’ stability and biodiversity. In Europe alone, this represents an estimated societal cost of 70–320 billion Euros per year [3]. Humankind’s intervention in the N cycle is considered to have already crossed the Earth’s ecological safety boundary, thus threatening our own security [2]. However, the demand for more food and energy due to increasing population and changing consumption patterns hampers the efforts of reducing N emissions [1].

Most of the observed increase in deposited N take two main forms: reduced N (NH_y_: NH_3_ and NH_4_^+^), primarily from volatilized agricultural emissions, and oxidized N (NO_x_: nitric acid, particulate nitrate, etc.), primarily from fossil fuel combustion. These different N forms can be deposited by both dry and wet deposition, in proportions that depend on their relative concentrations, precipitation patterns, and environmental drivers of biosphere-atmosphere exchange [4, 5]. Although an approximately 50% decrease in European NO_x_ deposition has been achieved since the 1980s, progress in reducing NH_y_ deposition has been much slower. This is especially true for the Iberian Peninsula, which has become a significant meat-exporting region [6, 7], an activity that releases high levels of reduced nitrogen through excrement and fertilizer used to produce animal feed. The ongoing revision of the EU National Emissions Ceilings Directive [8] proposes a 69% reduction in NO_x_ emissions and 27% reduction in NH_3_ emissions by 2030 compared with a 2005 baseline. In the case of Portugal, a 71% reduction in NO_x_ emissions is proposed, but only a 16% reduction in NH_3_ [8]. Thus, NH_3_ is expected to make a proportionately larger contribution to Portuguese N deposition in the future. In a global perspective, increased food demands and more fertilizer use may further increase agricultural N use [9] and NH_3_ emissions, while a combination of increased agricultural production and climate warming may lead to a doubling of global NH_3_ emissions by 2100 [10].

### Nitrogen pollution and biodiversity

#### Impacts of atN pollution on ecosystems and their biodiversity

Most natural terrestrial ecosystems have evolved under a specific and often low N availability, and thus can be changed by excessive N (i.e. atN pollution), through both direct and indirect mechanisms [11]. High levels of gaseous or aerosol-borne N (usually NH_3_) can be directly toxic to higher plants [12, 13] and to organisms that adsorb elements directly from the environment, such as algae, lichens, or bryophytes [14, 15]. Existing atN pollution also acts indirectly on organisms through factors such as nutrient enrichment, soil or water acidification, altered nutrient ratios, or by intensifying the impact of other stressors such as pathogens, herbivory or climate change [11]. Under atN pollution, species composition changes over time and diversity often declines, as characteristic species of oligotrophic, mesotrophic or circumneutral habitats (including species of conservation interest) are out-competed by faster-growing, more nitrophytic or acid-resistant plants, many of which are ruderals or invasive [11, 16]. In general, forbs, bryophytes, lichens and oligotrophic shrubs are the main functional types negatively affected by atN pollution, while grasses, adapted to higher nutrient levels, are the main functional type to benefit.

The impacts of atN pollution on biodiversity [15, 17–19] and on species’ physiology [20, 21] are a particular problem immediately downwind of sources such as intensive livestock production. Direct foliar damage is usually due to high local concentrations of NH_3_[11] while broader ecosystem-scale changes in soil and vegetation often result from chronically-elevated local and regional N deposition, including a combination of wet and dry deposition of NH_y_ and NO_x_ compounds [19, 22]. Within the soil, atN pollution can reduce the allocation of carbon from the vegetation to mycorrhizal fungi [23, 24] and other free-living microorganisms (e.g. other fungi, N-fixing bacteria, phosphorus solubilizers) thus impacting soil microbial communities and the ecosystem functions and services they provide (e.g. decomposition, biological N fixation). The atN pollution also impacts soil fauna [25]. N-driven changes in soil fauna and microbial communities influence the physical properties of soil, such as soil aggregation, water infiltration and organic matter turnover [26]. The impacts of atN pollution on a species or ecosystem depend on several factors [27], including the duration of exposure, total amount and form of N, species sensitivity, intrinsic ecosystem properties (e.g. fertility and acid neutralizing capacity) and climate [11].

Overall, atN pollution threatens biodiversity globally [11, 28], but a global analysis identified northern temperate, boreal, arctic, alpine, grassland, savannah and Mediterranean biomes as being particularly sensitive to atN pollution [28]. Biodiversity loss is of special concern in biodiversity hotspots such as Mediterranean type ecosystems [29, 30], which are thought to be experiencing the greatest proportional biodiversity change [28]. Of the five global Mediterranean regions (California, central Chile, Mediterranean Basin, southern Cape region and southwestern and southern Australia), California and the Mediterranean are considered those most threatened by atN pollution [31]. In contrast to Californian ecosystems, however, those in the Mediterranean Basin are still relatively poorly studied regarding the impacts of atN pollution [11, 31, 32].

#### Impacts of atN pollution on European habitats, including the Natura 2000 network

In this work, we focused on Natura 2000 areas because they host a significant portion of Europe’s biodiversity, including most of its sensitive and unique species. atN pollution constitutes a serious challenge for the conservation of such habitats and species under the Habitats Directive (92/43/EEC). The Habitats Directive, a cornerstone of Europe’s nature conservation policy, promotes the maintenance of biodiversity and requires the Member States to take measures to maintain or restore natural habitats at a favourable conservation status. The Directive established the Natura 2000 network with the aim of assuring the long-term survival of Europe’s most valuable and threatened species and habitats. These sites are afforded the highest degree of protection under European legislation: the provisions of the Directive require strict site protection measures, any avoidance of deterioration and a precautionary approach to permitting “plans or projects” which are likely to have a significant effect on a site. However, the Habitats Directive does not directly address air pollution impacts, of which N deposition and ozone are currently the most important, and until now, there has been no common European approach for determining the impacts of air pollution on individual sites or their conservation status [33].

To protect ecosystems from atN pollution, thresholds for N have been set as critical levels (atmospheric concentration) and critical loads (deposition in ecosystems) [34]. Exceedance of critical loads for N deposition is often associated with a reduction in plant species richness in a broad range of ecosystems. Critical loads of 5–10 kg N ha^-1^ yr^-1^ have been defined for sensitive ecosystems [24, 35], although there is evidence that individual sensitive species may decline at levels below the critical load [36], and effects may occur over the longer-term at lower loads [11]. Combining global modelled N deposition with the spatial distribution of protected areas under the UN Convention on Biological Diversity showed that 40% of all protected areas (or 11% of all ecosystems, by area) are projected to receive N deposition higher than 10 kg N ha^-1^ yr^-1^ by 2030 [37].

#### The Portuguese case

The Natura 2000 network together with the national network of protected areas covers approximately 22% of the mainland Portuguese terrestrial territory. Portuguese nature conservation areas are created and managed by the national authority for nature conservation ICNF (www.icnf.pt).

Emissions of NH_3_ are distributed unevenly throughout Portugal due to the patchy location of intensive livestock farming and agriculture, which comprise 85% of the total NH_3_ emissions. Some municipalities have relatively high emission densities due to the presence of point source emissions associated with industrial activities such as intensive pig and poultry rearing (6%). Overall, there has been a downward trend in NH_3_ emissions since 1990 (-22.7%), mainly due to decreasing numbers of cattle and energy production from renewable sources [38].

Only a few studies deal with the impact of atN pollution on Portuguese ecosystems. In a Mediterranean Basin matorral habitat (http://eunis.eea.europa.eu/habitats/1699), the form and dose of available N are being manipulated in an ongoing field experiment running since 2007. The study site, located south of Lisbon (Natura 2000 site PTCON0010 Arrábida/Espichel), has low ambient N deposition (<4 kg N ha^-1^ yr^-1^) and low soil N content (0.1%). N availability is increased in three N-treatments through additions of 40 kg N ha^-1^ yr^-1^ as a 1:1 NH_4_Cl to (NH_4_)_2_SO_4_ mixture, and 40 and 80 kg N ha^-1^ yr^-1^ as NH_4_NO_3_. The impacts on plant composition and diversity (richness and evenness) [16, 39] and ecosystem characteristics (soil extractable N and organic matter, aboveground biomass and % of bare soil) [16, 40] and functions (decomposition, nitrification, biological N fixation) [41] are assessed. In contrast to most similar studies, plant species richness increased with enhanced N input and was more related to ammonium than to nitrate. Data suggest that enhanced NH_4_^+^ availability affects the structure of the matorral, which may promote soil erosion and N leakage, whereas enhanced NO_x_ availability leads to biomass accumulation, which may increase fire risk [16]. Based on this experiment, the first empirical critical load of N for this European habitat was set at between 20–30 kg N ha^-1^ yr^-1^ [35].

Sclerophyllous grazed forests (*dehesas* in Spain and m*ontados* in Portugal - http://eunis.eea.europa.eu/habitats/10129) have been characterized regarding their NH_3_ critical levels and N critical loads [19], including for situations in which other pollution sources co-occur with N [22]. This was done using the changes in functional diversity of one of the most sensitive components of the ecosystem, epiphytic lichens [15]. Under atN pollution, the total plant species richness of these ecosystems did not change, but their functional diversity has undergone a complete shift from a community dominated by oligotrophic species to one dominated by nitrophytic ones [42]. This led to the establishment of a critical level for ammonia at 0.6 μgm^−3^ and a critical load for N at 26 kg ha^−1^ yr^−1^ [19], which is within the upper range established for other semi-natural ecosystems [19].

#### Aim

Taking into consideration the risk posed by N to biodiversity [43, 44], and the knowledge gaps identified for Portugal, the main objective of this work was to map the risk that atN pollution poses to changing biodiversity at Natura 2000 sites located in mainland Portugal. Because most areas with a conservation interest in Portugal occur within or in the immediate vicinity of agriculture fields, which emit mostly NH_x_, we have focused on NH_3_ emissions and used it as a proxy for overall atN pollution. We used the most recent spatially distributed NH_3_ emission inventory available for Portugal at the municipality level together with the location of the Natura 2000 sites. Then we ranked the sensitivity of the habitats within the Habitats Directive (92) to atN pollution using expert knowledge and produced a risk map integrating both NH_3_ emissions and Natura 2000 sites’ susceptibility to atN pollution. The risk map enables prioritisation of conservation strategies within the Natura 2000 network and identification of research gaps in evaluating the impacts of atN pollution on habitat conservation. In the following section, we review the ecological impacts of N deposition within the context of the EU Natura 2000 network and in relation to Portuguese conditions. Afterwards, we describe the assessment and mapping methodologies applied, and then present and discuss the results.

## Material and methods

We considered all Portuguese Natura 2000 sites where habitat information is publicly available (http://www.icnf.pt), resulting in a selection of 60 sites in mainland Portugal. Within these Natura 2000 sites, all habitats listed in Annex 1 of the Habitats Directive were considered for further analysis (88 habitats).

Only two of the habitats occurring in mainland Portugal have been studied regarding their sensitivity to atN pollution (see section 2.3). Therefore, the assessment of the sensitivity to atN pollution of the habitat types of Annex I of the Habitats Directive occurring in mainland Portugal was done by expert judgment. Seven experts were selected from professionals working in environmental companies and researchers working in biogeography, vegetation science, conservation and ecology with considerable experience of Portuguese habitats and an understanding of nitrogen pollution impacts. A higher number of experts was not possible since we required them to classify most of the habitats present in mainland Portugal, and only a restricted number of people have enough knowledge of such high number of habitats. Moreover, the impacts of nitrogen on biodiversity remain understudied in Portugal, making the choice of experts even narrower. Nevertheless, we are confident in the knowledge of the expert’s selected for this work. We are also confident that the results obtained are a major step up in current knowledge.

Each of the habitats in Table 1 was assessed by these experts regarding sensitivity to atN pollution on a score of 1 (least sensitive) to 10 (most sensitive). The N sensitivity of a given habitat was taken as the likelihood of biodiversity changes in response to atN pollution due to the proximity of an agricultural area. The average classification given by all experts (n = 7) was assumed as the habitat’s sensitivity to atN pollution, and the habitats were ranked accordingly. The relative standard deviations of the experts’ estimates are also shown (averaged per habitat), providing as a measure of uncertainty in the classification.

**Table 1:** Sensitivity of habitats designated under the Habitats Directive (92 - http://ec.europa.eu/environment/nature/legislation/habitatsdirective) to atN pollution and occurring in mainland Portugal.

		Habitat	N sensitivity
Coastal and halophytic habitats	Open sea and tidal areas	**1110** Sandbanks which are slightly covered by sea water all the time	2.6 0.44
		**1130** Estuaries	2.8 0.30
		**1140** Mudflats and sandflats not covered by seawater at low tide	2.6 0.44
		**1150** * Coastal lagoons	3.8 0.51
		**1160** Large shallow inlets and bays	3.6 0.32
		**1170** Reefs	4.0 0.64
	Sea cliffs and shingle or stony beaches	**1210** Annual vegetation of drift lines	3.0 0.51
		**1230** Vegetated sea cliffs of the Atlantic and Baltic coasts	3.5 0.47
		**1240** Vegetated sea cliffs of the Mediterranean coasts with endemic *Limonium* spp.	4.0 0.35
	Atlantic and continental salt marshes and salt meadows	**1310** *Salicornia* and other annuals colonizing mud and sand	4.0 0.32
		**1320** *Spartina* swards (*Spartinion maritimae*)	3.8 0.42
		**1330** Atlantic salt meadows (*Glauco-Puccinellietalia maritimae*)	4.0 0.42
	Mediterranean and thermo-Atlantic saltmarshes and salt meadows	**1410** Mediterranean salt meadows (*Juncetalia maritimi*)	4.0 0.42
		**1420** Mediterranean and thermo-Atlantic halophilous scrubs (*Sarcocornetea fruticosi*)	4.2 0.35
		**1430** Halo-nitrophilous scrubs (*Pegano-Salsoletea*)	4.2 0.49
	Salt and gypsum inland steppes	**1510** * Mediterranean salt steppes (*Limonietalia*)	3.2 0.46
	Sea dunes of the Atlantic, North Sea and Baltic coasts	**2110** Embryonic shifting dunes	4.8 0.48
		**2120** Shifting dunes along the shoreline with *Ammophila arenaria* (white dunes)	4.6 0.49
		**2130** * Fixed coastal dunes with herbaceous vegetation (grey dunes)	5.5 0.41
		**2150** * Atlantic decalcified fixed dunes (*Calluno-Ulicetea*)	6.0 0.35
		**2170** Dunes with *Salix repens* ssp. *argentea* (*Salicion arenariea*)	5.8 0.33
		**2180** Wooded dunes of the Atlantic, Continental and Boreal region	5.5 0.38
		**2190** Humid dune slacks	6.3 0.36
	Sea dunes of the Mediterranean coast	**2230** *Malcolmietalia* dune grasslands	3.7 0.48
		**2250** * Coastal dunes with *Juniperus* spp.	4.1 0.47
		**2260** *Cisto-Lavenduletalia* dune sclerophyllous scrubs	4.6 0.42
		**2270** * Wooded dunes with *Pinus pinea* and/or *Pinus pinaster*	4.4 0.41
	Inland dunes, old and decalcified	**2330** Inland dunes with open *Corynephorus* and *Agrostis* grasslands	4.7 0.32
Freshwater Habitats	Standing water	**3110** Oligotrophic waters containing very few minerals of sandy plains (*Littorelletalia uniflorae*)	8.1 0.17
		**3120** Oligotrophic waters containing very few minerals generally on sandy soils of the West Mediterranean with *Isoetes* spp.	8.1 0.17
		**3130** Oligotrophic to mesotrophic standing waters with vegetation of the *Littorelletea uniflorae* and/or *Isoeto-Nanojuncetea*	8.0 0.18
		**3140** Hard oligo-mesotrophic waters with benthic vegetation of *Chara* spp.	7.8 0.20
		**3150** Natural eutrophic lakes with *Magnopotamion* or *Hydrocharition*—type vegetation	5.1 0.52
		**3160** Natural dystrophic lakes and ponds	5.6 0.55
		**3170** * Mediterranean temporary ponds	7.1 0.28
	Running water	**3250** Constantly flowing Mediterranean rivers with *Glaucium flavum*	4.8 0.53
		**3260** Water courses of plain to montane levels with the *Ranunculion fluitantis* and *Callitricho-Batrachion* vegetation	5.8 0.39
		**3270** Rivers with muddy banks with *Chenopodion rubri* p.p. and *Bidention* p.p vegetation	4.3 0.23
		**3280** Constantly flowing Mediterranean rivers with *Paspalo-Agrostidion* species and hanging curtains of *Salix* and *Populus alba*	4.8 0.53
		**3290** Intermittently flowing Mediterranean rivers of the *Paspalo-Agrostidion*	4.8 0.53
Temperate heath and scrub		**4010** Northern Atlantic wet heaths with *Erica tetralix*	7.6 0.23
		**4020** * Temperate Atlantic wet heaths with *Erica ciliaris* and *Erica tetralix*	7.7 0.23
		**4030** European dry heaths	6.1 0.26
		**4060** Alpine and boreal heaths	6.5 0.29
		**4090** Endemic oro-Mediterranean heaths with gorse	5.8 0.43
Sclerophyllous scrub (matorral)	Sub-Mediterranean and temperate scrub	**5110** Stable xerothermophilous formations with *Buxus sempervirens* on rock slopes (*Berberidion* p.p.)	5.7 0.24
		**5120** Mountain *Cytisus purgans* formations	5.6 0.27
		**5140** * *Cistus palhinhae* formations on maritime wet heaths	5.9 0.33
	Mediterranean arborescent matorral	**5210** Arborescent matorral with *Juniperus* spp.	5.5 0.15
		**5230** * Arborescent matorral with *Laurus nobilis*	5.7 0.14
	Thermo-Mediterranean and pre-steppe brush	**5320** Low formations of *Euphorbia* close to cliffs	5.1 0.36
		**5330** Thermo-Mediterranean and pre-desert scrub	5.0 0.35
	Phrygana	**5410** West Mediterranean clifftop phryganas (*Astragalo-Plantaginetum subulatae*)	5.8 0.40
Natural and semi-natural grassland formations	Natural grasslands	**6110** * Rupicolous calcareous or basophilic grasslands of the *Alysso-Sedion albi*	4.2 0.38
		**6160** Oro-Iberian *Festuca indigesta* grasslands	4.3 0.45
	Semi-natural dry grasslands and scrubland facies	**6210** Semi-natural dry grasslands and scrubland facies on calcareous substrates (*Festuco-Brometalia*) (* important orchid sites)	6.3 0.18
		**6220** * Pseudo-steppe with grasses and annuals of the *Thero-Brachypodietea*	5.2 0.36
		**6230** * Species-rich Nardus grasslands, on siliceous substrates in mountain areas (and submountain areas, in Continental Europe)	6.2 0.22
	Sclerophillous grazed forests (dehesas)	**6310** Dehesas with evergreen *Quercus* spp.	5.3 0.26
	Semi-natural tall-herb humid meadows	**6410** *Molinia* meadows on calcareous, peaty or clayey-silt-laden soils (*Molinion caeruleae*)	6.8 0.22
		**6420** Mediterranean tall humid herb grasslands of the *Molinio-Holoschoenion*	5.8 0.14
		**6430** Hydrophilous tall herb fringe communities of plains and of the montane to alpine levels	5.8 0.17
	Mesophile grasslands	**6510** Lowland hay meadows (*Alopecurus pratensis*, *Sanguisorba officinalis*)	6.5 0.37
Raised bogs and mires and fens	*Sphagnum* acid bogs	**7140** Transition mires and quaking bogs	8.2 0.22
		**7150** Depressions on peat substrates of the *Rhynchosporion*	8.2 0.22
Rocky habitats and caves	Scree	**8130** Western Mediterranean and thermophilous scree	4.5 0.29
	Rocky slopes with chasmophytic vegetation	**8210** Calcareous rocky slopes with chasmophytic vegetation	5.0 0.35
		**8220** Siliceous rocky slopes with chasmophytic vegetation	5.4 0.36
		**8230** Siliceous rock with pioneer vegetation of the *Sedo-Scleranthion* or of the *Sedo albi-Veronicion dillenii*	5.4 0.36
		**8240** * Limestone pavements	5.0 0.35
	Other rocky habitats	**8310** Caves not open to the public	2.3 0.65
		**8330** Submerged or partially submerged sea caves	3.3 0.96
Forests	Forests of temperate Europe	**9160** Sub-Atlantic and medio-European oak or oak-hornbeam forests of the *Carpinion betuli*	6.2 0.24
		**91B0** Thermophilous *Fraxinus angustifolia* woods	5.4 0.32
		**91E0** * Alluvial forests with *Alnus glutinosa* and *Fraxinus excelsior* (*Alno-Padion*, *Alnion incanae*, *Salicion albae*)	6.5 0.23
		**91F0** Riparian mixed forests of *Quercus robur*, *Ulmus laevis* and *Ulmus minor*, *Fraxinus excelsior* or *Fraxinus angustifolia*, along the great rivers (*Ulmenion minoris*)	5.8 0.25
	Mediterranean deciduous forests	**9230** Galicio-Portuguese oak woods with *Quercus robur* and *Quercus pyrenaica*	6.3 0.29
		**9240** *Quercus faginea* and *Quercus canariensis* Iberian woods	6.3 0.33
		**9260** *Castanea sativa* woods	6.6 0.30
		**92A0** *Salix alba* and *Populus alba* galleries	6.5 0.32
		**92B0** Riparian formations on intermittent Mediterranean water courses with *Rhododendron ponticum*, *Salix* and others	7.0 0.30
		**92D0** Southern riparian galleries and thickets (*Nerio-Tamaricetea* and *Securinegion tinctoriae*)	5.9 0.39
	Mediterranean sclerophyllous forests	**9320** *Olea* and *Ceratonia* forests	6.0 0.26
		**9330** *Quercus suber* forests	6.3 0.33
		**9340** *Quercus ilex* and *Quercus rotundifolia* forests	6.2 0.31
		**9380** Forests of *Ilex aquifolium*	7.2 0.18
	Mediterranean and Macaronesian mountainous coniferous forests	**9560** * Endemic forests with *Juniperus* spp.	6.5 0.27
		**9580** * Mediterranean *Taxus baccata* woods	6.7 0.26

Sensitivity to atN pollution was assessed by expert judgement (average and relative standard deviation; n = 7 experts) ranging from 1 to 10, with 10 corresponding to the most N sensitive habitat and 1 to the least N sensitive habitat.

* indicates priority habitat types according to the European Union Habitats Directive.

We considered as the habitat’s susceptibility the potential adverse effects caused by atN pollution to the organisms living in each Natura 2000 Portuguese sites. Then, all Natura2000 sites were classified according to their susceptibility to atN pollution, considering the average of their habitats’ sensitivity to atN pollution.

The NH_3_ emissions reported by the Portuguese authorities (http://www.apambiente.pt/) were used as a proxy for atN pollution (including atmospheric concentrations and dry deposition of ammonia). This is feasible because NH_3_ is generally deposited close to its emission sources [45–47]. The most recent NH_3_ emission data available at the municipality level (year 2009) were spatially disaggregated to the area occupied by each Natura 2000 site. Then the average NH_3_ emissions were calculated for each Natura 2000 site.

Finally, we considered environmental risk as "the combination of the probability, or frequency, of occurrence of a defined hazard and the magnitude of the consequences of the occurrence" [48]. Combining each site’s susceptibility to atN pollution with the estimated local NH_3_ emissions enabled assessment of the relative risk of biodiversity change occurring for each Natura 2000 site, due to atN pollution. The maximum risk was defined as the combination of highest NH_3_ emissions and highest susceptibility to atN pollution; in total, nine classes of risk were defined.

## Results & discussion

**Fig 1:**
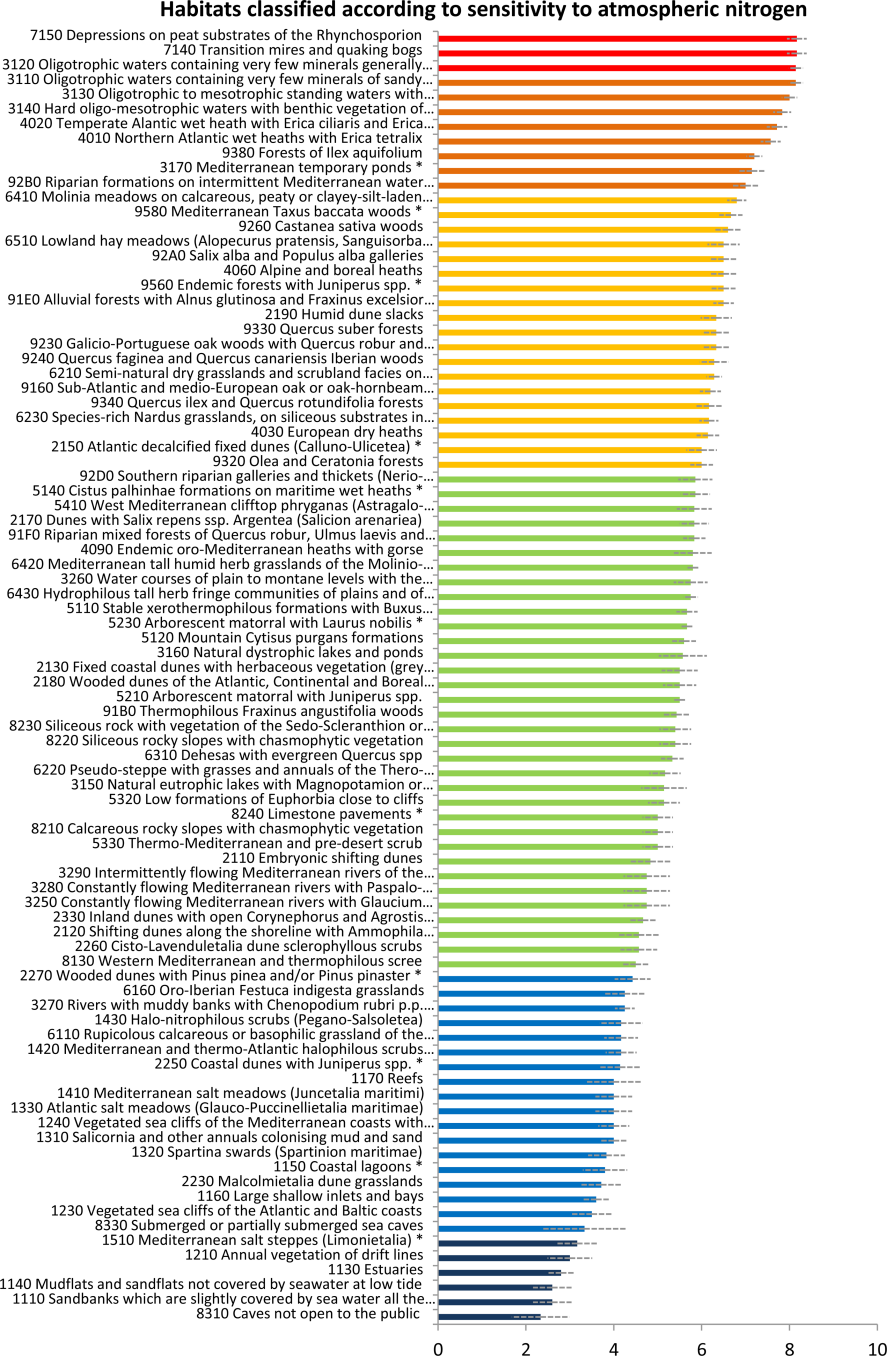
Ranking of the habitats (Habitats Directive) occurring in mainland Portugal according to their sensitivity to atmospheric Nitrogen pollution (higher values in red indicate higher sensitivity). The bars represent the average relative standard deviation (the same as in Table 1). See full habitat names in Table 1.

Of the 88 habitat types defined for Natura 2000 sites across mainland Portugal (Fig 1), 37 occur in the Atlantic region, 81 in the Mediterranean region and 36 in both (see S1 File). Furthermore, from the 121 plant species of Community Importance, 117 occur in the Mediterranean region, and 27 in the Atlantic region, thus making the Mediterranean region biologically ‘richer’ than the Atlantic one for conservation purposes. Table 1 shows all habitats classified according to their sensitivity to atN pollution, with average expert ranking ranging from 2.3 to 8.2 (higher values representing the most sensitive habitats). Ranking the habitats’ sensitivity to atN pollution (Fig 1) gave that the most sensitive habitats as:

i) ‘Raised bogs and mires and fens–*Sphagnum* acid bogs’, particularly habitat **7140**—Transition mires and quaking bogs and habitat **7150**—Depressions on peat substrates of the *Rhynchosporion*;
ii) ‘Freshwater Habitats—Standing water’, particularly habitat **3130**—Oligotrophic to mesotrophic standing waters with *Littorelletea uniflorae* and/or *Isoete Nanojuncetea*, habitat **3110**—Oligotrophic waters containing very few minerals of sandy plains (*Littorelletalia uniflorae*) and habitat **3120**—Oligotrophic waters containing very few minerals generally on sandy soils of the West Mediterranean with *Isoetes* spp.

The least sensitive habitats were:

i) ‘Rocky habitats and caves–Other rocky habitats’, particularly habitat **8310**—Caves not open to the public;
ii) ‘Coastal and halophytic habitats—Open sea and tidal areas’, particularly habitat **1110**—Sandbanks which are slightly covered by sea water all the time, habitat **1140**—Mudflats and sandflats not covered by seawater at low tide and habitat **1130**—Estuaries.

The most sensitive habitats were acidic and oligotrophic while the least sensitive were coastal open sea, tidal habitats and caves, which often house large bat colonies that emit large amounts of NH_3_ through the accumulation of guano in the caves.

**Fig 2:**
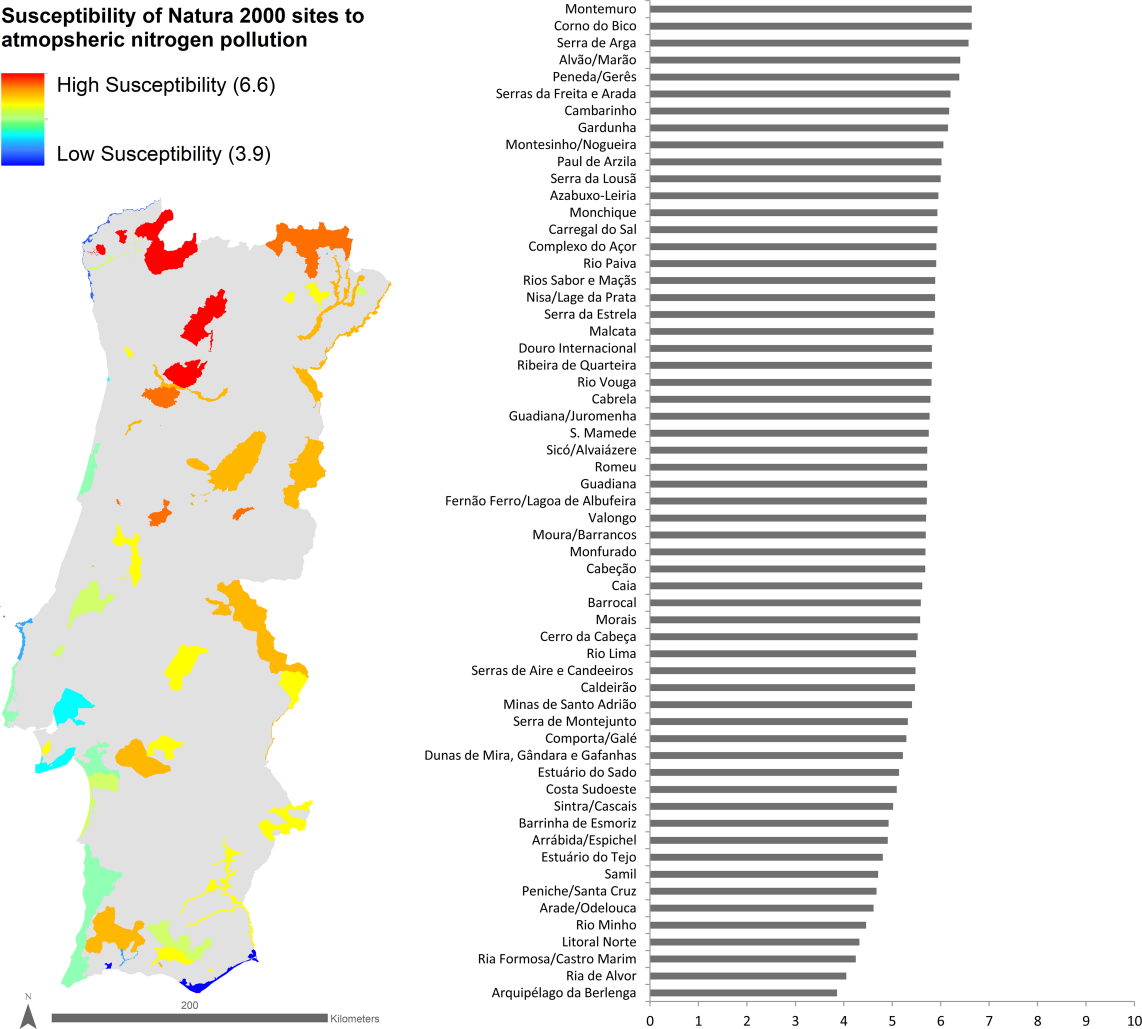
Map and plot of Natura 2000 sites in mainland Portugal, classified according to their susceptibility to atmospheric Nitrogen pollution. Higher values indicate higher susceptibility, on a scale from 1 to 10.

Distributing the 88 habitats along the 60 Natura 2000 sites in which they occur enabled mapping the susceptibility to atN pollution for each site (Fig 2). The sites’ susceptibility to atN pollution ranged from 6.6 at the most susceptible site to 3.9 at the least susceptible site. Sites located inland in the northern mountains were considered to be more susceptible to atN pollution than the sites located along the coast. Sites located inland in the Mediterranean region of mainland Portugal showed intermediate scores of susceptibility to atN pollution. Examples of the Natura 2000 sites most susceptible to atN pollution included PTCON0039 (Serra de Arga), PTCON0040 (Côrno do Bico) and PTCON0025 (Montemuro), while examples of the least susceptible to atN pollution included PTCON0006 (Arquipélago da Berlenga), PTCON0058 (Ria de Alvor) and PTCON0013 (Ria Formosa/Castro Marim).

**Fig 3:**
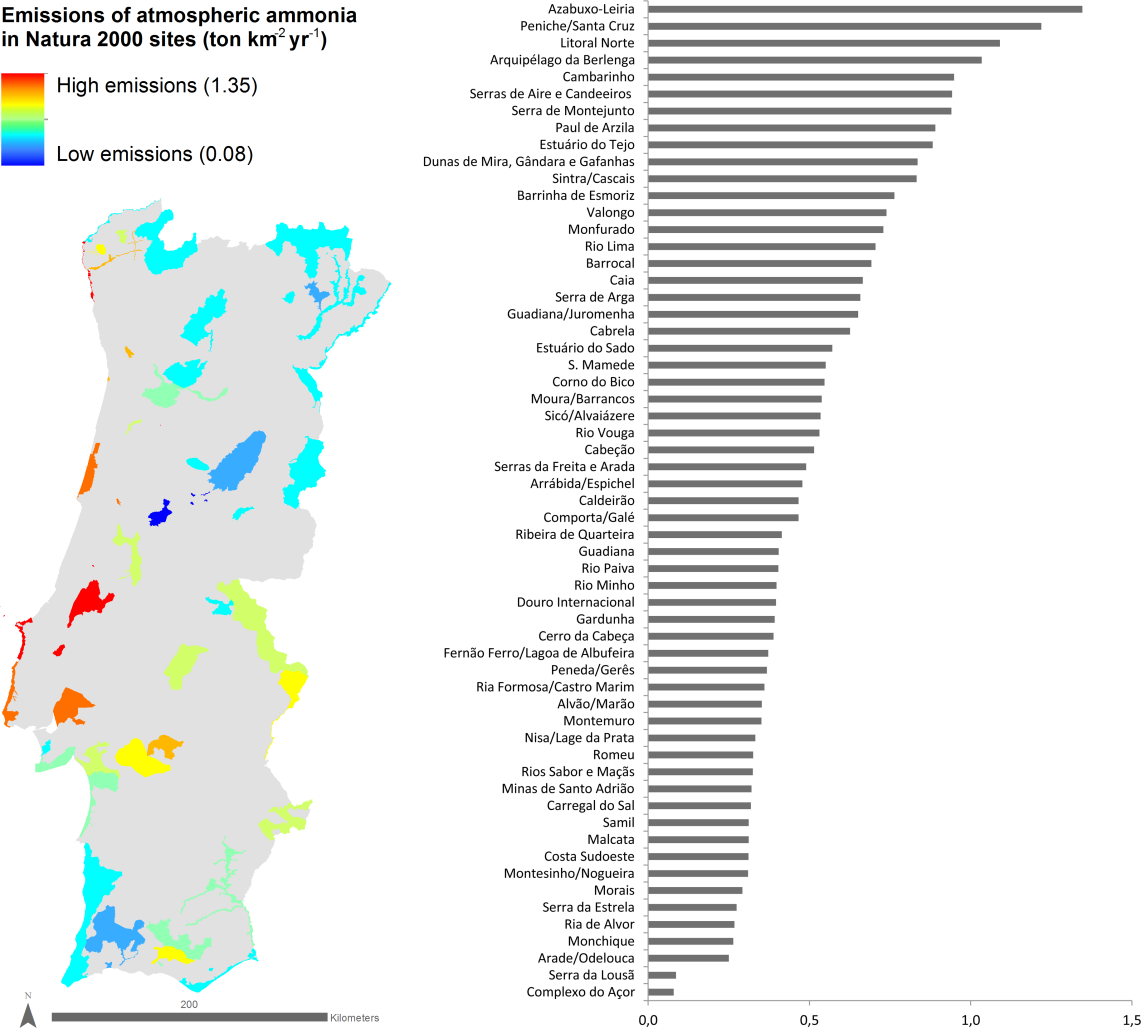
Map and plot of Natura 2000 sites in mainland Portugal, classified according to the average atmospheric ammonia emissions (2009, tonne km^-2^ year^-1^).

Given that the Natura 2000 network and protected areas occupy ca. 22% of the area of mainland Portugal, and that the distribution of the Portuguese population and anthropogenic activities is not uniform across the territory, several Natura 2000 sites are in close proximity to important sources of NH_3_ pollution. Such sites near intensive agricultural areas were within grid squares with the highest NH_3_ emissions (and so were assumed to have high NH_3_ concentrations), in contrast to more remote sites (Fig 3). PTCON0006 (Arquipélago da Berlenga), PTCON0017 (Litoral Norte), PTCON0056 (Peniche/Santa Cruz) and PTCON0046 (Azabuxo- Leiria) had NH_3_ emissions > 1 ton km^-2^ yr^-1^ while PTCON0051 (Complexo do Açor) and PTCON0060 (Serra da Lousã) had NH_3_ emissions one order of magnitude smaller (< 0.1 ton km^-2^ yr^-1^).

**Fig 4:**
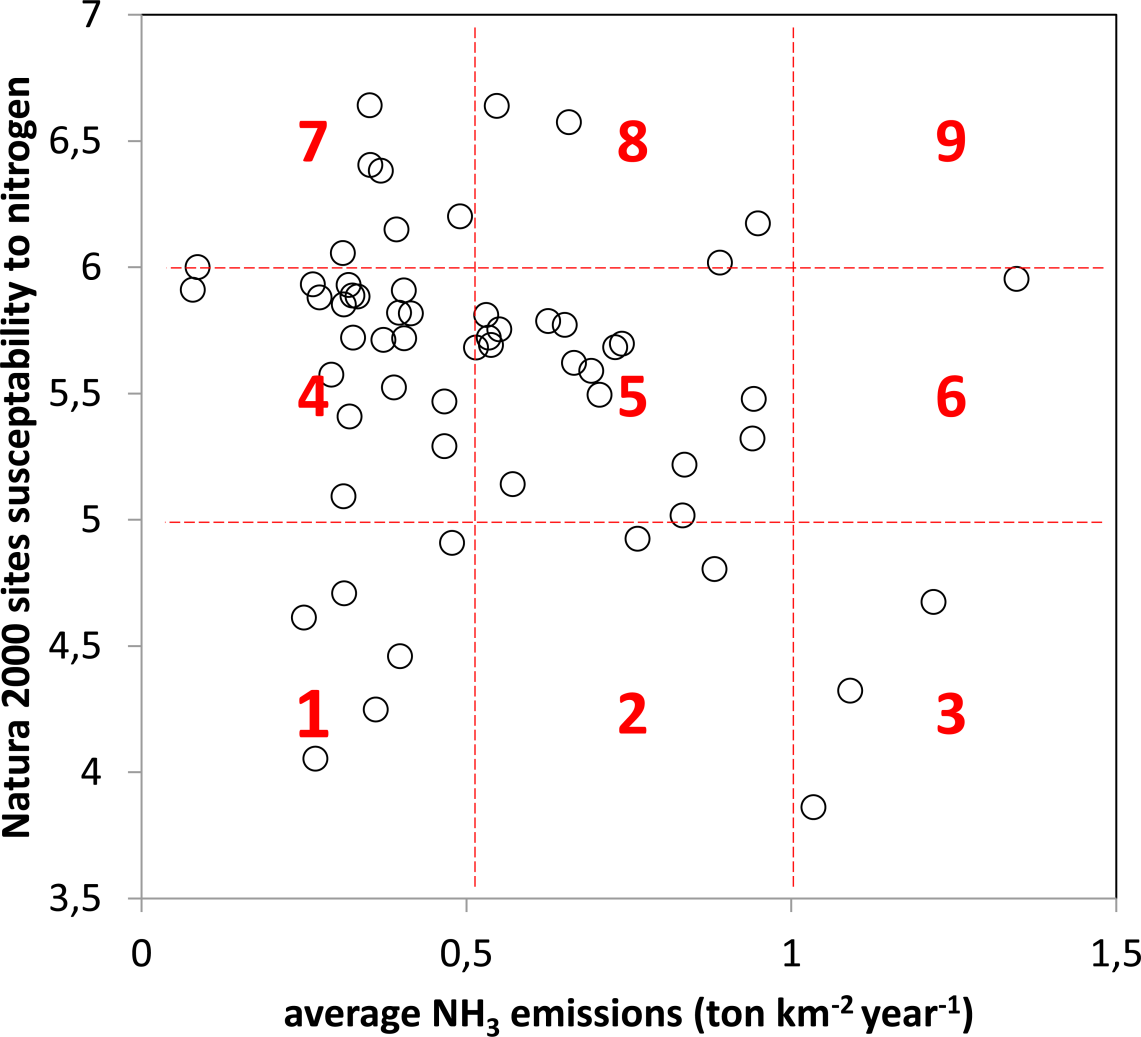
Plot of Natura 2000 sites’ susceptibility to atmospheric nitrogen (atN) pollution vs. average ammonia emissions, showing the relative risk of biodiversity change due to atN pollution. It is notable that there are no Natura 2000 sites in the highest risk category (9) of highest susceptibility to atN pollution and highest ammonia emissions, which may reflect some degree of spatial separation between agricultural activities and natural habitats.

**Fig 5:**
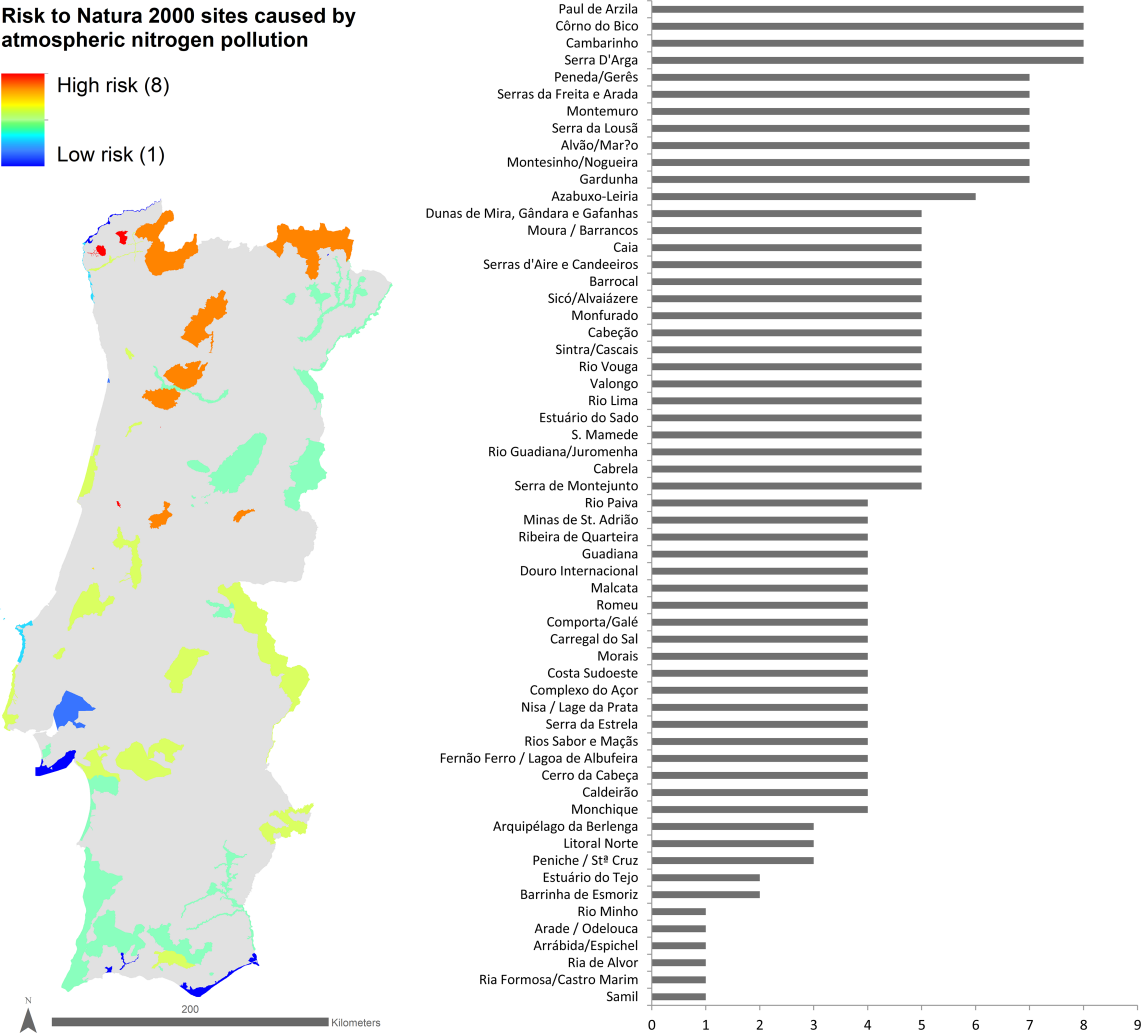
Map and plot of Natura 2000 sites classified according to the relative risk of biodiversity change caused by atmospheric Nitrogen (atN) pollution. Higher values indicate higher risk, which is caused by a combination of site’s high susceptibility to atN pollution and high emission of ammonia.

The combined analysis of the Natura 2000 sites’ susceptibility to atN pollution and NH_3_ emissions was used to calculate the relative risk of habitat change caused by atN pollution (Figs 4 and 5 and S1 File). No Natura 2000 site was found to fall into the maximum risk category (9), i.e. a combination of the highest susceptibility to atN pollution and highest NH_3_ emissions (Fig 4). Four sites were considered to be in the second-highest risk category (8): Paúl de Arzila, Côrno do Bico, Cambarinho and Serra d’Arga. The sites with the lowest risk (category 1) were Rio Minho, Arade/Odelouca, Arrábida/Espichel, Ria de Alvor, Ria Formosa/ Castro Marim and Samil. In general, the Natura 2000 sites with the lower relative risk of biodiversity change due to atN pollution were either coastal or dominated by Mediterranean evergreen species (Matorral and semi-natural grassland formations). Most coastal sites have saltmarshes, which are thought to cope relatively well with atN pollution because their naturally low aerobic conditions make ammonia the predominant N form [49]. Sites dominated by evergreen Mediterranean oaks, which dominate most of the southern part of mainland Portugal, are already adapted to some degree of disturbance, i.e. mainly low-intensity agriculture and grazing [50]. These ecosystems were shaped over millennia, and their current species pool–including those of conservation interest–reflect that long-term process, by showing a lower susceptibility to atN pollution and thus an overall lower relative risk of biodiversity change due to atN pollution.

The Natura 2000 sites located further north are dominated by deciduous Mediterranean oaks and showed higher risk of biodiversity change due to atN pollution than the southern sites. This is likely associated with granitic soil in these areas, and the presence of the most N sensitive habitat types, i.e. peatland, mires and bogs. These habitats were not shaped by low-level agricultural activities and their soil (more acidic) offer less buffering capacity to atN pollution. Although studies have shown that the species richness of bogs is less sensitive to N deposition than other oligotrophic habitats such as acid grassland or heathland [51, 52] (which are not represented in the Natura 2000 sites), this is because bog vegetation, particularly *Sphagnum*, can shift from pollution-sensitive species to less sensitive ones as pollution increases [52, 53], resulting in comparatively little change in species richness but a significant change in community composition.

In this study, we only mapped the Natura 2000 sites at higher relative risk of biodiversity change due to atN pollution taking NH3 as a proxy for overall atN pollution. Other forms of N could be considered, namely NOx. However, NOx is only relevant for a few sites located near large urban or industrial areas and in general, reduced N (ammonia/ammonium) has been shown to cause more negative impacts than similar doses of oxidized N [16, 54, 55]. Altogether, NH_3_ emission can be regarded as a surrogate of overall atN pollution on each site, because it is the prevailing N form emitted in the airsheds of Portuguese Natura 2000 sites. In our study, all habitats were treated as being of the same conservation value. Further studies could investigate ranking habitat value within the Ecosystem Services framework [56], by including which services would be lost or gained if a given habitat disappeared due to atN pollution. In this work we focused in atmospheric nitrogen. However, for habitats greatly influenced by running water, the main threats may come from surface runoff of nitrogen rich water. Thus, those habitats risk of change would have increased if we could consider such nitrogen source. However, that information is not available, and thus this work represents the best approach possible to those habitats susceptibility to nitrogen pollution.

This study relied on the relative classification of all Natura 2000 in mainland Portugal using expert knowledge. Thus, we stress that we obtained the relative position of sites, not an absolute valuing of their sensitivity. Moreover, this study is limited by the use of expert knowledge. However, this is currently the only approach available to define research and action priorities regarding atN pollution. The alternative option would be to establish the empirical critical loads for N of the habitats present in all Natura 2000 sites, and if those critical loads are being exceeded. However, this type of knowledge is not available for the vast majority of the Portuguese mainland ecosystem (but see [40] [41]). Thus, the use of expert knowledge is currently the only option available for this study. Although seven experts may seem a small number of replicates, we could only consider those experts with knowledge on most habitats and on N issues, and that combination of skills is not frequent. One way to take into account the uncertainty of the responses of experts is to consider the habitat classification deviation (see standard deviation values in Table 1). This deviation was not very high (average relative standard deviation average = 0.35) reflecting that experts coincided in general in their appraisal. However, some habitats presented rather conflicting evaluations, with a relative standard deviation of 0.55 or higher, namely 3160 (Natural dystrophic lakes and ponds), 1170 (Reefs), 8310 (Caves not open to the public) and 8330 (Submerged or partially submerged sea caves). Among the other habitats with high deviation, we found mostly marine and aquatic habitats, which probably reflect the poorer knowledge available for those habitat types.

## Conclusions

In this study, we combined mainland Portuguese NH_3_ emission estimates with an expert-based classification of Natura 2000 habitats’ sensitivity to atN pollution. This allowed us to calculate and map the relative risk of biodiversity change in Natura 2000 sites due to atN pollution. Results identified mountain habitats with mires, fens and bogs as the most sensitive to atN pollution, while coastal salt marshes were considered the least sensitive. By combining habitat sensitivity with local NH_3_ emissions, we identified the Natura 2000 sites in mainland Portugal that are at higher relative risk of biodiversity change due to atN pollution: these are mostly located in the northern mountains, close to areas with agricultural activities of moderate intensity.

This ranking approach can be used to prioritize conservation efforts to those sites most at risk. The most sensitive Portuguese habitats (bogs, mires) are relatively widespread in temperate northern Europe, and the impacts of atN pollution on these types of ecosystems have been previously evaluated [11, 35, 51]. It remains an open question to what extent the sensitivity of these habitats in the warm Atlantic context of Portugal is different from Northern Europe. More importantly, very little is still known about the impacts of long-term elevated atN pollution on Mediterranean habitats that, in Europe, only occur in the south, and constitute major biodiversity hotspots which are considered a global conservation priority [29, 30]. Therefore, and despite their lower current estimated susceptibility to atN pollution, we consider that these Mediterranean habitats’ uniqueness and a large contribution to Europe’s biodiversity and natural capital make them a priority in terms of improving future understanding the impacts of atN pollution. Understanding the role of heterogeneity, patchiness and disturbance over millennia on the resilience of these habitats [57] may also be a key to better management [58] and to building resilience in the most sensitive habitats.

## Supporting information

S1 File Final map as kml (google earth). Map showing all Natura 2000 sites in mainland Portugal classified according to the relative risk from nitrogen pollution. Blue represent the lowest potential risk, red represents the highest risk.
(7Z) Click here for additional data file.
